# Exercise training improves vascular function and secondary health measures in survivors of pediatric oncology related cerebral insult

**DOI:** 10.1371/journal.pone.0201449

**Published:** 2018-08-09

**Authors:** Treya M. Long, Shoshana R. Rath, Karen E. Wallman, Erin K. Howie, Leon M. Straker, Andrew Bullock, Thomas S. Walwyn, Nicholas G. Gottardo, Catherine H. Cole, Catherine S. Choong, Louise H. Naylor

**Affiliations:** 1 School of Human Sciences, Exercise and Sport Science, The University of Western Australia, Perth, Western Australia, Australia; 2 Department of Endocrinology, Perth Children’s Hospital, Perth, Western Australia, Australia; 3 School of Medicine, Pediatrics, The University of Western Australia, Perth, Western Australia, Australia; 4 School of Physiotherapy and Exercise Sciences, Curtin University, Perth, Western Australia, Australia; 5 Department of Cardiology, Perth Children’s Hospital, Perth, Western Australia, Australia; 6 Department of Hematology and Oncology, Perth Children’s Hospital, Perth, Western Australia, Australia; 7 Telethon Kids Institute, Perth, Western Australia, Australia; 8 Hematology, PathWest Laboratory Services, Perth, Western Australia, Australia; Universidad Europea de Madrid, SPAIN

## Abstract

Adolescent and young adult (AYA) survivors of pediatric oncology related cerebral insult are vulnerable to numerous treatment-induced deficits that significantly enhance cardiovascular disease risk. Regular exercise improves endothelial function, fitness, body composition and musculoskeletal function which may reduce predisposition for cardiovascular disease. Here we assessed the feasibility and effectiveness of a 24-week exercise intervention on cardiovascular, physical and metabolic outcomes in this population. Thirteen survivors (6 male, 7 female; median age 19 y (range 16–23 y) were recruited to participate in a 48-week study consisting of a 24-week control period (regular care) followed by a 24-week exercise intervention. Outcome measures were collected at entry (week 0) and following regular care (24-week) and exercise (48-week). Assessed variables included endothelial function (flow mediated dilation, FMD), blood pressure, heart rate (HR), aerobic capacity, anthropometry, body composition, muscular strength (3 repetition maximum testing), muscular endurance (repetitions/min) and physical activity levels (accelerometry). Compared to baseline, delta diameter (p = 0.008) and FMD (p = 0.029) of the brachial artery increased following exercise. Bicep-curl strength also increased following exercise compared to baseline (p = 0.019), while submaximal (6 min mark) measures of ventilation (p = 0.012), rating of perceived exertion (p = 0.012), HR (p = 0.001), absolute (p = 0.000) and relative (p = 0.000) aerobic capacity decreased. Breaks in sedentary time increased (p = 0.043) following exercise compared to regular care. Although the sample was small and heterogeneous, this study demonstrates that exercise is achievable and has positive effects on vascular function, submaximal fitness, local strength and physical activity in a population of AYA survivors of pediatric oncology related cerebral insult.

## Introduction

Advances in treatment have increased the population of pediatric cancer survivors over the past three decades, with approximate five-year survival rates in Australia, America and the United Kingdom currently sitting at 85% [1–3]. However, concurrent with improved survival is the eventual development of an array of long-term and late side-effects with potentially dire health consequences [1, 4, 5]. As a result, the incidences of chronic disease and early mortality among this population are in excess of the normal population [1, 4, 6, 7]. Indeed, cardiovascular events are currently the number one non-malignant cause of mortality among pediatric cancer survivors [6, 7].

Survivors of pediatric oncology related cerebral insult are among those at the highest risk of developing severe, disabling or life-threatening long-term and late effects [1, 4]. The invasive nature of cancer and its treatment during childhood may limit physical performance and exacerbate cardiovascular disease (CVD) risk during adolescence and young adulthood [8–11]. Cardiotoxicity is a well-known side effect of certain chemotherapeutic agents, most notably the anthracyclines [12–14]. Specifically, the anthracyclines have direct toxic effects on the vascular endothelium and cardiac myocytes, with a dose dependent relationship resulting in clinical heart failure at cumulative doses > 550mg/m^2^ [15]. Radiation to the heart, young age at exposure and concomitant therapy with agents such as cyclophosphamide further increase risk of cardiotoxicity, which increases linearly as a function of follow-up time [15]. In addition, radiotherapy can cause fibrosis and atrophy of muscular tissues, thereby inhibiting function of the cardiac, vascular and/or musculoskeletal structures within the area of exposure [16, 17]. Accelerated atherosclerosis has also been reported in vessels within the radiation portal, which predisposes to myocardial infarction and stroke [18, 19]. Although radiation dosage to the heart is lower in this cohort than for those with Hodgkin’s Lymphoma or solid tumors, cardiac fibrosis and ventricular dysfunction are potential late complications that may result from exit radiation in patients receiving craniospinal irradiation for intracranial tumors such as medulloblastoma. Notably, our group and others have also previously demonstrated that CVD may develop secondary to the direct ramifications of cancer and its treatment [20]. For example, hypothalamic insult instigates disorders of endocrine function, energy balance and metabolism, which manifest as obesity, cachexia and fatigue [5, 20, 21]. Subsequently, limitations to activities necessary for basic care and health maintenance may develop, exacerbating CVD risk by means of physical de-conditioning [4, 5, 20, 22, 23].

While these limitations are well recognized, few studies have attempted to minimize or reverse them using an intervention. Exercise improves global function of the cardiovascular, respiratory, musculoskeletal and metabolic systems [24–26]. It also encourages breaks in prolonged bouts of sedentary time, which is preventative against deterioration [27, 28]. As a result, we propose that exercise may be a potential therapy for the prevention and/or remediation of long-term and late effects that predispose cancer survivor populations to accelerated CVD. Both Fiuza-Luces et al. [29] and San Juan et al. [30] demonstrated that exercise during pediatric cancer therapy has positive effects on body strength, aerobic fitness and functional mobility. To date, the few studies that have utilized exercise interventions in survivor populations post-therapy have also reported positive outcomes, with reports of improved exercise tolerance, maximal aerobic performance, upper and lower body strength, function, quality of life and reduced fatigue [31, 32]. However, the majority of these exercise training studies have been performed in survivors of leukemia or breast cancer, with little evidence regarding efficacy in pediatric cancer survivors who have sustained a brain insult and suffer specific long-term morbidity. As such, it is unknown whether these side-effects will limit the extent of the benefits gained from regular exercise. Here, we implemented a 24-week exercise intervention in adolescent and young adult (AYA) survivors of pediatric oncology related cerebral insult. Endothelial function, a surrogate measure of CVD [33, 34], was assessed before and after intervention to ascertain effectiveness of exercise in ameliorating cardiovascular risk in this population. Secondary risk factors for CVD that were measured included aerobic capacity, resting heart rate (HR) and blood pressure (BP), physical activity (PA) levels, anthropometry, body composition and muscular strength and endurance.

## Methods

### Participants

A search of the Princess Margaret Hospital for Children (Western Australia) oncology database identified 87 long-term (>5 years) AYA (15–23 years) survivors of pediatric oncology related cerebral insult that were eligible to participate in the study. Survivors were contacted about participation via phone and mail. Thirteen (6 male, 7 female) survivors consented to participate and met all study requirements to be included in analysis. Seven additional survivors participated in the study to varying degrees but were excluded from the final results due to drop out (n = 2; both cases related to relapse) or non-compliance to the intervention standards (n = 5; work and university timetables clashed with the exercise sessions making regular attendance difficult).

Pregnancy or a diagnosis of CVD were exclusion criteria from this study. Four of our participants were growth hormone deficient (GHD) and had previously been treated with GH. Three ceased treatment six months prior to study participation, while one started low-dose replacement therapy following baseline assessment. Survivors taking thyroid (n = 2) and sex hormone (n = 4) supplements were not excluded. Prior to participation survivors were informed of all study requirements and were asked to provide written consent. All survivors below the age of 18 were required to have written parental or guardian consent for participation. Ethical approval was granted by the University of Western Australia (UWA) Human Research Ethics Committee and the Princess Margaret Hospital Ethics Committee (HREC approval number, 2013059.).

### Experimental design

Upon recruitment, resting BP, HR, endothelial function, anthropometry, body composition, muscular strength and endurance and aerobic capacity were assessed. Accelerometers and activity journals were used to record physical activity levels over a seven day period.

Following baseline testing participants began a 24-week control phase (regular care) during which they were instructed to maintain usual PA levels and habits. Immediately following the regular care phase a 24-week exercise intervention was commenced. Order was non-randomized to counter potential carry-over effects and difficulties employing extended wash-out periods. At the conclusion of the 24-week exercise intervention all assessments were repeated. To account for a lack of healthy controls in this study, we have compared our “Following Exercise” survivor data to baseline data collected on a similarly aged, healthy control group from a previous characterization publication [20] in the discussion of this manuscript.

### Vascular function

On arrival to the laboratory, following a four hour fasting period, participants rested supine for 20 min. During this rest HR, BP and mean arterial pressure (MAP) were measured at five min intervals using an electronic BP cuff (HR and BP; Dinamap Carescape V100, GE Healthcare, General Electric’s Company, Buckinghamshire, UK).

Following rest, conduit artery function was assessed using the flow mediated dilation (FMD) technique as detailed by Thijssen et al. [35]. Non-invasive, high-resolution ultrasound (Terason, t3200, Burlington, MA 01803, USA) was used to image the left brachial artery. Brachial artery diameter was recorded for one min before a forearm cuff placed distal to the olecranon process was inflated to 220 mmHg for five min. Diameter and blood flow recordings resumed 30 sec before cuff deflation and continued for three min. Brachial artery blood flow was calculated using measurements of continuous (30 Hz) brachial lumen cross-sectional area and Doppler velocity. Change in artery diameter between baseline and peak was presented in mm (delta diameter). Flow mediated dilation (delta percent) was calculated as (delta diameter/baseline diameter) x 100. Specific details of FMD analysis and analysis software have been presented elsewhere [35].

### Physical activity monitoring

Participants were fitted with an Actical accelerometer (Respironics; Bend, Oregon, USA) to record PA during waking hours over seven days. The Actical accelerometer was chosen for its lightweight (28×27×10 mm: 17 g), waterproof design, and omnidirectional sensor [36]. As per the manufacturer’s instructions, accelerometers were placed medial to the right iliac crest (under clothing), fastened by an adjustable waist strap [37]. Participants were provided with an activity journal and were instructed to record daily accelerometer use, as well as activity undertaken whilst wearing the accelerometer. Accelerometers were not worn during the exercise sessions to ensure recordings were of external and volitional PA.

To encourage compliance, participants received thrice-weekly text messages and phone calls (upon receipt of the monitor, mid-week and prior to monitor return) each time they were in possession of the Acticals. This ensured that participants were wearing the monitors and that journal recordings were being made.

### Anthropometry and body composition

Anthropometric measurements included body-mass, height and body-mass index (BMI). Body-mass was rounded to the nearest 0.01 kilogram (kg) using an electronic scale (Sauter Model EB60, FSE Scientific, New South Wales, AUS) and a wall-mounted stadiometer (Seca 216 Measuring Pole, Birmingham, UK) measured height to the nearest tenth of a centimeter. Body-mass index was calculated as body-mass (kg) divided by height squared, as defined by The World Health Organization (WHO) [38].

Body composition was assessed using dual x-ray absorptiometry (DXA; Lunar iDXA, GE Healthcare, Madison, USA). Total body and peripheral fat were presented in kg and as percentages of tissue mass, while visceral adipose tissue (VAT) was presented in terms of mass (g) and volume (cm^3^). Total lean body mass (LBM) was presented in kg.

### Muscular strength and endurance

Protocols for muscular strength and endurance testing were in accordance with the American College of Sports Medicine (ACSM) guidelines for exercise testing and prescription [26] and are detailed by Long et al. [20]. Three repetition maximum (3RM) testing was used as a clinically safe, yet accurate [26, 39], way to assess latissimus dorsi pull-down and bicep curl strength. This assessment has previously been used in a pediatric brain cancer survivor population with no adverse events [20] and none occurred during testing for the current study. Strength testing was limited to the upper body to avoid causing or exacerbating any hip or knee joint pain in our population. To account for this, muscular endurance of the trunk and lower body (squats, sit-ups and push-ups) was assessed by determining the maximum number of repetitions participants could complete of each exercise in 60 sec.

### Aerobic capacity

Submaximal (3 min, 6 min and 9 min) and peak (**V**O_2peak_) aerobic capacity was assessed on a treadmill using a modified chronotropic protocol designed for clinical populations. Procedural details can be found in Long et al. [20].

Incremental stages were three min in duration and participants were encouraged to continue until volitional exhaustion. Heart rate (Polar Electro Oy, Professorintie Kempele, Finland), BP (Bronze Series DS54 DuraShock Hand Aneroid Sphygmomanometer, Welch Allyn, New York, USA) and ratings of perceived exhaustion (RPE) based upon the Borg scale (6–20) [40] were recorded in the last 30 sec of each stage. Heart rate and BP were also recorded pre and post assessment.

Expired air was collected through a mouthpiece connected to a computerized gas analysis system. Measurements of minute ventilation (V_E_) and respiratory exchange ratio (RER) were recorded every 15 sec. Aerobic capacity at both submaximal and peak stages were recorded in absolute (L∙min^-1^) and relative (ml·kg^-1^·min^-1^) terms. End-stage absolute VO_2peak_ was also converted into ml and divided by LBM for a true representation of peak aerobic capacity.

### Exercise intervention

Exercise sessions were 90 min long and held three days per week. Participants were required to attend a minimum of two sessions per week in order to be included in the final analysis. Those able to attend all three training sessions were strongly encouraged to; however, there was low adherence at the third session which was held on a weekend. To aid adherence, the program was held at a number of venues chosen for their proximity to the participant’s home addresses. Attendance was marked at each session and participants contacted via phone if they appeared to be falling behind and had not given previous notice of absence.

Exercise programs were designed by an accredited exercise physiologist and followed ACSM [26] guidelines for exercise prescription. These programs were tailored for each individual based on their baseline strength, endurance and aerobic capacity testing results and included elements of both resistance and aerobic training. All programs were monitored during each exercise session to ensure adherence; the supervising exercise physiologist marked off exercises on each individual’s training log as they were completed, and monitored intensity of each using the Borg scale (6–20) [40]. A session was considered completed if ≥90% of the prescribed exercises had been performed within the time frame.

Resistance work encompassed all major muscle groups (chest, abdomen, back, shoulders, arms and legs; ~6–10 exercises) and was performed in a circuit with short rest periods (3–5 min) between exercises. A combination of free weights (eg. bicep curls, tricep extensions, shoulder press), machine weights (eg. leg curl, leg extension, chest press, latissimus dorsi pull down) and body weight (eg. squats, calf raises, abdominal curls, bridges) was used to provide a range of resistance levels and exercise types. While weight was individualized (60–70% of 3RM; ~50–60% of 1RM), sets and repetitions were generally kept constant between participants (3 sets, 10 repetitions). Programs were progressed by increasing weight every 3–4 weeks, or earlier if self-reported and/or observed effort reached light to moderate (11–13) on the Borg scale (6–20) [40].

Aerobic training utilized a moderate intensity interval protocol whereby sub-maximal sprints (~60% HR maximum) were alternated with periods of active rest (~40% HR maximum). Intensity was monitored throughout using RPE and HR readings provided by hand sensor pads on the ergometers. Participants performed four consecutive sprint-rest bouts before passively resting for 3–5 minutes. This was repeated three times using different ergometers (rower, stationary bike, arm cycler). Total time of aerobic activity for all participants was between 10–15 min. Progressions were made by first decreasing time of active rest and then by increasing sprint duration. This occurred whenever self-reported and/or observed effort fell below ~11 on the Borg scale (6–20) [40], or when the participant had been on the same protocol for 3–4 weeks.

### Data analyses

Three days (two weekdays and one weekend, ≥ 8 hr) of full recorded accelerometer data from each participant was analyzed. Participants were excluded from analysis if they lost or failed to return their accelerometer, or if wear time was too sporadic. Actograms were visually inspected to remove any sleep and/or non-wear time from the data, before the Freedson equation [41] and a translation equation by Straker and Campbell [36] were used to process the data in LabView V7® (National Instruments, Austin, Texas, USA). Sedentary time was measured using a threshold of 91 counts per min (cpm) [36, 41]. Thresholds for light, moderate and vigorous intensity PA were 1776 cpm, 5180 cpm and >5180 cpm, respectively [36, 41]. Breaks were defined as a disturbance in sedentary behavior that registered > 100 cpm and lasted at least one min. Physical activity guidelines [24–26] were used for comparison to assess whether participants were meeting daily PA recommendations (at least 30 min of daily, moderate intensity PA).

### Statistical analyses

Data was analyzed using SPSS version 20.0 (IBM, USA). One-way repeated measures ANOVA’s were applied to identify changes in measures over time, with significance set at p ≤ 0.05. Where appropriate, paired t-tests were used to make post hoc comparisons between assessment points. Cohen’s D effect sizes [42] were calculated for all measures, between all time-points. Only moderate (0.5–0.79) and large (≥ 0.8) effect sizes are reported. All descriptive data is presented as mean ± standard deviation (SD).

In order to assess whether nature of the tumors had any effect on our results a sub-analysis occurred whereby survivor data was further categorized into benign and malignant groups, and then re-analyzed using mixed model ANOVA’s. Significance was set at p ≤ 0.05 and post hoc comparison occurred as above. Due to the large amount of additional data this analysis procured, and to avoid repeating time effects, only significant group differences have been presented in text. Remaining data from this analysis can be found elsewhere (S1 Table.).

## Results

### Participant characteristics

As a group, median age of the participants was 19 y (range 16–23 y), while median age at diagnosis was 3 y (range 3 months– 10 y) Thus, median time since diagnosis was 15 y (range 7–22 y), while median time since final treatment was 13 y (range 7–21 y).

Median age of benign tumor survivors was 19 y (range 16–21 y). Median age at diagnosis was 3 y (range 9 months– 10 y), median time since diagnosis was 15 y (range 7–20 y), while median time since final treatment was 11 y (range 7–19 y). Malignant tumor survivors had a median age of 19 y (range 19–23 y). Median age at diagnosis was 3 y (range 3 months– 5 y). Median time since diagnosis was 16 y (range 14–22 y), while median time since final treatment was 13 y (range 12–21 y). Additional treatment and diagnostic information is presented in Table 1.

**Table 1:** Participant characteristics.

	Survivors (n = 13)	
	No.	%
Underlying Diagnosis		
Brain Tumor	9	69.23
Tumor Type		
Craniopharyngioma	1	7.69
Glioma	7	53.85
Medulloblastoma	1	7.69
Tumor Location		
Brain Stem	1	7.69
Frontal Lobe	2	15.39
Optic Pathway	1	7.69
Posterior Fossa	2	15.39
Subependymal Zone	1	7.69
Temporal Lobe	2	15.39
Leukemia	3	23.08
ALL	3	23.08
Other	1	7.69
Undifferentiated rhabdomyosarcoma of the right petrous temporal bone	1	7.69
Treatment		
Surgery	5	38.46
Surgery & XRT	1	7.69
Chemotherapy & XRT	1	7.69
Surgery, Chemotherapy & XRT	4	30.77
Chemotherapy, XRT & HSCT	2	15.39
Treatment Details		
XRT	8	61.54
Dosage		
6–24 Gy	3	23.08
50–56 Gy	5	38.46
Location		
Cranial	3	23.08
Craniospinal	3	23.08
Total Body	2	15.39
Chemotherapy	7	53.85
Agents		
Alkylating Agents	7	53.85
Anthracyclines	4	30.77
Vinca Alkaloids	6	46.15
Age at First Exposure	8	
<5 years	6	46.15
6–10 years	2	15.39
Age at Last Exposure	8	
<5 years	2	15.39
6–10 years	4	30.76
11–15 years	2	15.39
Other Characteristics		
Growth Hormone Deficiency	4	30.77

ALL, acute lymphoblastic leukemia; HSCT, hematopoietic stem cell transplant; XRT, radiotherapy

### Vascular function

At baseline, mean resting HR was 71 ± 8 bpm, mean SBP was 111 ± 10, and mean DBP was 64 ± 3.There were no changes in HR (74 ± 12 and 76 ± 16, following regular care and exercise respectively, p = 0.535), SBP (113 ± 11 and 116 ± 14, following regular care and exercise respectively, p = 0.148), or DBP (63 ± 4 and 63 ± 6, following regular care and exercise respectively, p = 0.587) over the course of the study.

**Fig 1:**
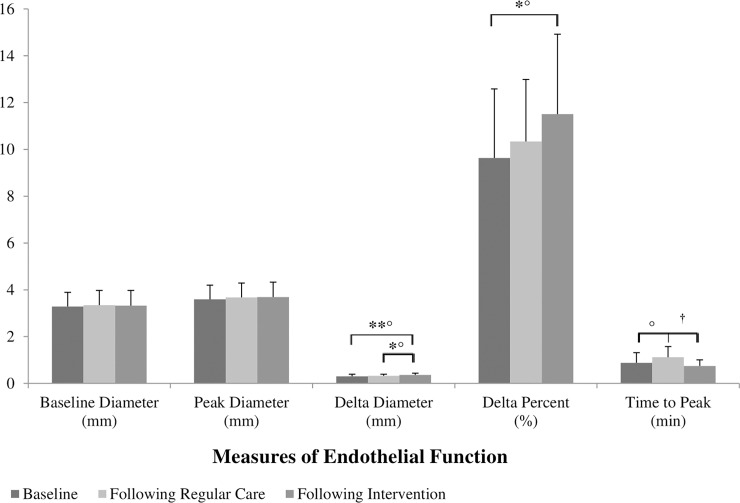
Change in endothelial function over the course of the study. *Baseline*: *n = 13*, *Following Regular Care*: *n = 13*, *Following Exercise*, *n = 13*, *p < 0.05, **p < 0.01, °*d > 0*.*5*, ^*†*^*d < -0*.*8*.

Endothelial function data is presented in Fig 1. A significant change in delta diameter and FMD was found over the course of the study (p = 0.006 and p = 0.031, respectively). Compared to baseline, delta diameter and FMD increased following the exercise intervention (p = 0.008, *d* = 0.72 and p = 0.029, *d* = 0.63 respectively). Delta diameter was also elevated following exercise when compared to regular care (p = 0.043, *d* = 0.56). There was a moderate, positive effect size (*d* = 0.56) suggesting that time to peak increased following regular care when compared to baseline. A large, negative effect size (*d* = -0.83) indicates time to peak shortened again following exercise when compared to regular care. Baseline and peak diameter remained unchanged across all time points.

With regards to endothelial function differences between benign and malignant tumor survivors, the malignant group had reduced times to peak dilation (S1 Table.). There were no other group differences present.

### Physical activity monitoring

**Fig 2:**
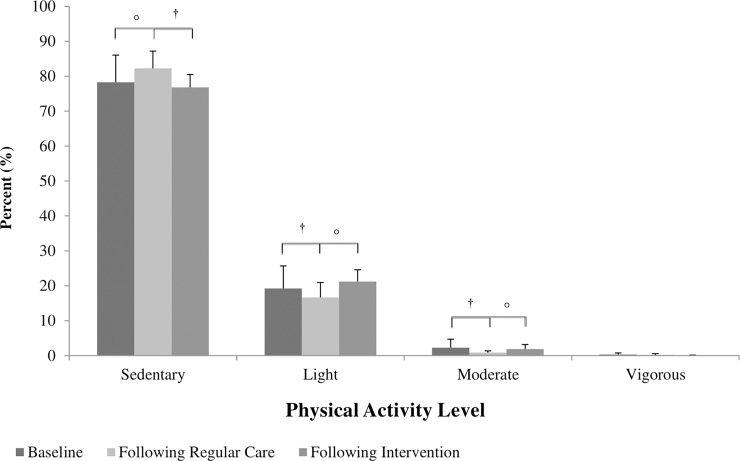
Percentage of time per day spent in different levels of physical activity. Baseline: n = 13, Following Regular Care: n = 9, Following Exercise, n = 10, ^†^d < -0.5, °d > 0.60.

Accelerometer wear time was highest at baseline when compared to regular care (Table 2; p = 0.002) and exercise periods (p = 0.028). There was a positive effect of exercise on a decline in volitional PA found to occur during regular care. Compared to baseline, breaks in sedentary time decreased following regular care (Table 2; p = 0.021, *d* = -0.79). Accelerometer counts also decreased following regular care compared to baseline (Table 2; *d* = -0.56), while percentage of sedentary time subsequently increased (Fig 2; *d* = 0.68), albeit both were insignificant. This cycle of physical inactivity was reversed with exercise training, with number of breaks and counts increasing from regular care (p = 0.043, *d* = 1.14 and *d* = 0.63, respectively) and sedentary time decreasing (*d* = -1.01). Percentage of time spent in light and moderate intensity PA followed similar patterns (Fig 2), although these were also statistically insignificant. Compared to baseline, percentages of light (*d* = -0.61) and moderate (*d* = -0.5) intensity PA showed tendencies to decline following regular care; however, both improved again following exercise when compared to regular care (light intensity, *d* = 0.96; moderate intensity, *d* = 1.86).

**Table 2:** Accelerometer, body composition and muscular strength and endurance data.

		Baseline		Following Regular Care		Following Exercise		
		Mean	SD	Mean	SD	Mean	SD	*P* value
Accelerometer Data							
N	13		9		10		
Wear Time (min/day)	842.60	21.63	752.34°^a^	10.74	767.54^†^^b^	18.32	0.003*
Sedentary Breaks	62.43	19.36	50.64°^a^	13.44	68.13^Δ^^c^	14.26	0.048*
Counts (per min)	164.92	121.38	109.72^a^	53.01	147.37^c^	46.39	0.222
Body Composition							
N	13		13		13		
Total Fat Mass (kg)	23.76	15.16	24.56	14.93	23.23	15.75	0.559
Total Percent Fat (%)	35.17	11.25	35.48	12.26	33.46	12.81	0.178
VAT Mass (g)	594.44	742.96	626.44	615.47	397.91	260.20	0.316
VAT Volume (cm^3^)	630.33	787.46	664.22	652.24	421.91	275.85	0.316
Total Lean Body Mass (kg)	40.62	12.80	41.38	13.07	41.99	13.28	0.202
Muscular Strength (kg)							
N	13		13		13		
Latissimus Dorsi Pull Downs	41	17	39	20	40	19	0.730
Bicep Curl	7.5	2.5	8.0	3.0	9.0^†^^Δ^	3.5	0.009*
Muscular Endurance (60 s)							
N	13		13		13		
Squats	33	15	30	11	32	12	0.409
Sit-ups	26	11	28	11	28	11	0.643
Push-ups	23	12	21	10	27^c^	14	0.180

VAT, visceral adipose tissue

*Denotes statistical significance (p ≤ 0.05) between time points using One-Way Repeated Measures ANOVA

°Denotes statistical significance (p ≤ 0.05) between Baseline and Regular Care using post-hoc paired t-tests

^†^Denotes statistical significance (p ≤ 0.05) between Baseline and Exercise using post-hoc paired t-tests

^Δ^Denotes statistical significance (p ≤ 0.05) between Regular Care and Exercise using post-hoc paired t-tests

^a^Denotes moderate to large effect size (*d* = ≥ 0.5) between Baseline and Regular Care using Cohen’s D effect sizes

^b^Denotes moderate to large effect size (*d* = ≥ 0.5) between Baseline and Exercise using Cohen’s D effect sizes

^c^Denotes moderate to large effect size (*d* = ≥ 0.5) between Regular Care and Exercise using Cohen’s D effect sizes

With regards to PA recommendations, mean moderate intensity PA was 19.20 ± 23.57 min/day at baseline, indicating that this cohort were inactive upon entry [24–26]. Physical activity did not reach the recommended levels over the course of the study (6.59 ± 3.97 min/day following regular care and 13.90 ± 9.46 min/day following intervention). Examination of individual data indicated only one participant met the daily activity recommendations following intervention (35.00 min/day).

### Anthropometry and body composition

Upon entry, average height was 164.5 ± 12.6 cm, body-mass was 67.86 ± 24.65 kg and BMI was 24.8 ± 8.2. There were no significant changes in body-mass (following regular care, 67.70 ± 24.04 kg; following exercise, 68.33 ± 24.33 kg; p = 0.707) or BMI (following regular care, 24.8 ± 8.4; following exercise, 25.2 ± 8.8; p = 0.435) over time. There were no significant effects or effect sizes for any of the DXA measures (Table 2). Further, the malignant tumor survivors were shorter in stature, and had reduced body mass and lean body mass compared to benign tumor survivors (S1 Table.).

### Muscular strength and endurance

There was a significant change in bicep curl strength over time (Table 2); in particular, an increase following exercise was apparent compared to both baseline (p = 0.019) and regular care (p = 0.041). There was a moderate, positive effect size for push-ups after the exercise intervention compared to regular care (*d* = 0.54). No main effects or effect sizes were observed for latissimus dorsi pull-downs, squats or sit-ups.

### Aerobic capacity

Aerobic capacity data can be found in Table 3. With regards to the 3 min submaximal epochs recorded during the VO_2peak_ test, there were significant main effects for V_E_, RER, absolute and relative VO_2peak_. Following regular care these measures were reduced compared to baseline (V_E_, p = 0.011, *d* = -0.71; RER, p = 0.016, *d* = -0.68; absolute VO_2peak_, p = 0.010, *d* = -0.97; relative VO_2peak_, p = 0.011, *d* = -2.26). Following exercise, V_E_ (p = 0.015, *d* = 0.57), RER (p = 0.017, *d* = 1.83) and relative VO_2peak_ (p = 0.025) increased compared to regular care. Further, absolute and relative VO_2peak_ decreased after exercise compared to baseline (p = 0.004, *d* = -0.91 and *d* = -1.37, respectively). At the first interval of the exercise test (3 min mark), submaximal HR and RPE was found to be lower after exercise compared to baseline (*d* = -0.53 and *d* = -0.5, respectively). There were significant changes recorded during the second interval of the exercise test (6 min mark) for all in submaximal aerobic measures over time. Minute ventilation, absolute and relative VO_2peak_ decreased following regular care (V_E_, p = 0.007, *d* = -0.54; absolute VO_2peak_, p = 0.036; relative VO_2peak_, p = 0.036, *d* = -0.81) and following exercise (V_E,_ p = 0.012, *d* = -0.58; absolute VO_2peak_, p = 0.000, *d* = -0.93; relative VO_2peak_, p = 0.000, *d* = -1.35) compared to baseline. Further, absolute VO_2peak_ declined after exercise compared to regular care (p = 0.012), with relative VO_2peak_ following similar patterns (*d* = -0.56). Also at this 6 min interval, RER decreased following regular care compared to baseline (p = 0.018, *d* = -0.66) and increased again following exercise (p = 0.000, *d* = 1.22) when compared to regular care. Heart rate for this epoch was lowest following exercise (compared to baseline, p = 0.001, *d* = -0.88 and following regular care, p = 0.034, *d* = -0.5), as was RPE (compared to baseline, p = 0.012, *d* = -0.5 and following regular care, *d* = -0.52). For the 9 min time point, V_E_, absolute and relative VO_2peak_ all decreased following regular care (V_E_, p = 0.000, *d* = -0.6; absolute VO_2peak_, p = 0.015, *d* = -0.69; relative VO_2peak_, p = 0.010, *d* = -0.78) and following exercise (V_E_, p = 0.006, *d* = -0.67; absolute VO_2peak_, p = 0.001, *d* = -0.94; relative VO_2peak_, p = 0.001, *d* = -1.32) compared to baseline. Submaximal 9 min HR was also lower following exercise compared to baseline (p = 0.001, *d* = -0.78). With regards to peak aerobic data, there was a main effect for RER. Notably, RER following regular care was significantly reduced compared to baseline (p = 0.035, *d* = -0.88) and following exercise (p = 0.001, *d* = 1.12).

**Table 3:** Submaximal and maximal aerobic capacity data.

	Baseline		Following Regular Care		Following Exercise		
	Mean	SD	Mean	SD	Mean	SD	*P* value
Aerobic Capacity (3 min Stage)							
N	13		13		13		
Rating Perceived Exertion	8	2	8	2	7^b^^c^	1	0.252
Submaximal HR (bpm)	104	10	103	14	99^b^	13	0.281
Minute Ventilation (L·min^-1^)	23.59	8.67	17.44°^a^	5.97	20.83^Δ^^c^	4.71	0.004*
Respiratory Exchange Ratio	0.79	0.27	0.61°^a^	0.07	0.72^Δ^^c^	0.04	0.042*
VO_2_ (L·min^-1^)	1.13	0.30	0.83°^a^	0.40	0.86^†^^b^	0.29	0.004*
VO_2_ (ml·kg^-1^·min^-1^)	15.84	1.70	12.05°^a^	3.08	13.51^Δ^^b^	2.55	0.010*
Aerobic Capacity (6 min Stage)							
N	13		13		13		
Rating Perceived Exertion	9	2	9	2	8^†^^b^^c^	2	0.025*
Submaximal HR (bpm)	137	17	132	20	122^†^^Δ^^b^^c^	15	0.001*
Minute Ventilation (L·min^-1^)	28.45	8.47	23.89°^a^	8.09	23.53^†^^b^	6.23	0.007*
Respiratory Exchange Ratio	0.86	0.29	0.67°^a^	0.09	0.78^Δ^^c^	0.04	0.048*
VO_2_ (L·min^-1^)	1.35	0.34	1.15°	0.45	0.98^†^^Δ^^b^	0.31	0.000*
VO_2_ (ml·kg^-1^·min^-1^)	18.79	2.27	16.66°^a^	2.48	15.26^†^^b^^c^	2.07	0.001*
Aerobic Capacity (9 min Stage)							
N	13		13		13		
Rating Perceived Exertion	11	2	10^a^	2	9^b^^c^	2	0.142
Submaximal HR (bpm)	161	12	156	14	152^†^^b^	12	0.002*
Minute Ventilation (L·min^-1^)	35.64	12.03	28.41°^a^	9.15	27.57^†^^b^	7.56	0.000*
Respiratory Exchange Ratio	0.95	0.43	0.72^a^	0.08	0.80^c^	0.03	0.098
VO_2_ (L·min^-1^)	1.63	0.52	1.27°^a^	0.58	1.14^†^^b^	0.42	0.001*
VO_2_ (ml·kg^-1^·min^-1^)	22.69	4.10	19.52°^a^	2.63	17.30^†^^Δ^^b^^c^	2.42	0.000*
Aerobic Capacity (Peak)							
N	13		13		13		
Rating Perceived Exertion	17	2	16	2	17	3	0.319
Maximal Heart Rate (bpm)	184	10	180	16	181	15	0.201
Minute Ventilation (L·min^-1^)	73.18	30.86	62.44	23.16	68.85	22.29	0.114
Respiratory Exchange Ratio	0.98	0.08	0.91°^a^	0.09	1.01^Δ^^c^	0.08	0.003*
VO_2peak_ (L·min^-1^)	2.72	1.03	2.46	1.10	2.51	1.14	0.163
VO_2peak_ (ml·kg^-1^·min^-1^)	38.50	12.32	37.35	11.94	38.53	12.92	0.431
VO_2peak_ (ml·LBM^-1^·min^-1^)	61.07	10.04	60.74	10.91	58.25	11.25	0.261

HR, heart rate; LBM, lean body mass.

*Denotes statistical significance (p ≤ 0.05) between time points using One-Way Repeated Measures ANOVA

°Denotes statistical significance (p ≤ 0.05) between Baseline and Regular Care using post-hoc paired t-tests

^†^Denotes statistical significance (p ≤ 0.05) between Baseline and Exercise using post-hoc paired t-tests

^Δ^Denotes statistical significance (p ≤ 0.05) between Regular Care and Exercise using post-hoc paired t-tests

^a^Denotes moderate to large effect size (*d* = ≥ 0.5) between Baseline and Regular Care using Cohen’s D effect sizes

^b^Denotes moderate to large effect size (*d* = ≥ 0.5) between Baseline and Exercise using Cohen’s D effect sizes

^c^Denotes moderate to large effect size (*d* = ≥ 0.5) between Regular Care and Exercise using Cohen’s D effect sizes

Differences in absolute aerobic capacity between benign and malignant tumor survivors were evident at all sub-maximal stages (S1 Table.). There were no other group differences present.

## Discussion

This study assessed the effect of a 24-week exercise intervention on CVD risk in AYA survivors of pediatric oncology related cerebral insult. In our cohort, exercise training induced significant improvements in endothelial function, submaximal aerobic capacity, PA levels and local strength.

Impairment of endothelial function precedes morphological changes that contribute to atherosclerotic lesion development and progression of CVD [34, 35]. Radiotherapy has been shown to accelerate plaque deposition, while certain classes of chemotherapeutic agents (eg. alkylating agents, anthracyclines, antiangiogenics) are reported to cause direct injury to the endothelium [13, 14, 18, 19]. We observed improvements in delta diameter, FMD and time to peak in brachial artery function after exercise training. Notably, the FMD in survivors after exercise was comparable to the FMD reported at baseline in a group of similarly aged healthy controls (11.51% vs. 10.18%)[20]. Importantly, an increase in FMD corresponds to a decreased risk of cardiac events [33, 43, 44]. This is a compelling finding and, to our knowledge, the first of its kind in this specific population. Järvelä et al. [12] reported an increase in brachial FMD following 16-weeks of home exercise in AYA survivors of pediatric leukemia; however, recordings were not continuous and changes in FMD were only apparent at 40 sec post cuff release. In contrast, our study involved continuous recordings and demonstrated improvements across the entire protocol. This suggests that exercise plays an important role in alleviating endothelial dysfunction in AYA survivors of pediatric oncology related cerebral insult. Additionally, it establishes that exercise can help to normalize endothelial function in survivors. When analyzing survivor data based on nature of the tumor, the malignant survivors had reduced times to peak dilation. As there are no other endothelial differences between these groups, and time to peak is not a useful adjunctive measure of endothelial function [35], the relevance of this finding is limited.

In contrast to the majority of previous reports, we did not observe any changes in peak aerobic capacity with exercise [11, 32]. As a result, peak aerobic capacity remained lower than it was in healthy controls from our previous publication (38.53 ml·kg^-1^·min^-1^ vs. 46.61 ml·kg^-1^·min^-1^) [20]. We theorize that late-effects exclusive to oncology related cerebral insult survivors (eg. GHD) may limit the extent of cardiorespiratory benefits gained from exercise, although it is beyond the scope of our study to determine this. Fitness may have also been impacted by adherence issues and the subsequent reduction in number of exercise sessions held per week. Interestingly, endothelial benefits seen here occurred irrespective of changes in end-stage VO_2peak_. This is another novel finding in this population and parallels vascular and aerobic capacity data reported in adolescents with type II diabetes [45]. Importantly, we observed significant improvements in submaximal aerobic capacity after exercise training. Participation in activities necessary for daily living (ADL’s), employment, homemaking, socialization and basic care require an ability to maintain prolonged, submaximal aerobic workloads [46]. However, AYA survivors of pediatric oncology related cerebral insult may suffer cardiorespiratory deconditioning secondary to direct consequences of treatment [4, 5, 20, 22, 23]. During the 6 min submaximal epoch of the VO_2peak_ exercise test our participants were working at 40–60% of their HR reserves, which is classified as moderate intensity activity according to ACSM [26]. This corresponds to 3–6 metabolic equivalents–the energy cost required to perform most household chores and ADL’s [47]. Thus, our data revealed that exercise is effective at improving submaximal aerobic capacity and, hence, ability to perform necessary daily tasks in this population. While other studies have reported improvements in peak or maximal aerobic capacity in pediatric cancer survivors [11, 32], our results instead align with those presented by Keats and Culos-Reed [48] and Piscione et al. [49], who found improvements in submaximal fitness among pediatric brain cancer cohorts.

Although the survivor strength and endurance measures after exercise were lower than those reported in healthy controls at baseline [20], we observed an improvement in bicep curl strength. There was also a moderate influence of exercise on push-up endurance. These results are consistent with previous research [30, 48, 50] and indicate an improved ability of our survivors to perform tasks requiring the use of the upper-limb, which may further ease ADL’s and help improve quality of life. With respect to body composition, we discovered no significant effects of exercise on adiposity. As a result, survivors had higher levels of fat than controls at baseline [20]. Nonetheless, improvements in endothelial function indicate that cardiovascular health and disease risk can be ameliorated in this population irrespective of changes in fat mass. Of note, the survivors of malignant tumors were shorter in stature than the benign tumor survivors and had reduced body masses and lean body masses. Based on previous literature [20, 51–53], we hypothesize that these anthropometric differences are due to the spinal and total body irradiation received exclusively by the malignant tumor survivors. These body mass differences subsequently affected absolute aerobic capacity between benign and malignant groups at submaximal stages.

Health guidelines recommend 30 min of daily, moderate intensity PA to protect against CVD [24, 26]. Additionally, increasing breaks in sedentary time is associated with improved cardiovascular, metabolic and physical profile independent of PA levels [27, 28].

Compared to normative data reported by Colley et al. [54], the survivors here recorded higher percentages of sedentary time (average 79% vs. 62%) and less light (average 20% vs. 29%) and moderate-to-vigorous (average 0.9% vs. 6%) intensity PA compared to healthy adolescents. Further, when compared to healthy adults aged 20–39 years, our survivors recorded fewer minutes of moderate intensity PA per day at all time points (baseline, 19.20 min/day; following regular care, 6.59 min/day; following exercise, 13.9 min/day vs. 24 min/day) [55]. This indicates a substantial risk for inactivity induced disease among our cohort. However, while the survivors here failed to increase their amount of volitional PA (ie. PA external to what was undertaken in the study exercise sessions) to meet recommendations throughout the course of the study, we observed a significant increase in sedentary breaks following the exercise intervention. Further, the downward spiral of inactivity and deconditioning that ensued regular care was arrested as a result of exercise. Keats and Culos-Reed [48] reported comparable findings regarding the immobilizing effect of exercise on physical inactivity in adolescent cancer patients. This highlights the importance of exercise interventions for influencing positive PA behaviors and for further reducing CVD risk in this population. Of note, accelerometer wear time was reduced compared to baseline at the regular care and exercise time points. We believe that assessment fatigue was the primary reason for these differences; that is, participants became bored or tired with wearing the monitors each day and subsequently forgot, or refused, to continue wearing them. Despite these differences, significant improvements in activity measures were evident, indicating the positive effect of exercise on PA behaviors.

Our results do not vary greatly compared to other exercise intervention studies implemented in pediatric cancer populations. Both Fiuza-Luces et al. [29] and Piscione et al. [49] also observed limited improvements following the implementation of exercise interventions in pediatric patients and survivors with solid tumors. Similar to findings here, Fiuza-Luces et al. [29] failed to see improvements in VO_2peak_, body mass and BMI following a thrice-weekly exercise program held over 19 weeks. However, there was a significant improvement in 5RM global body strength that we failed to find [29]. Conversely, we observed improvements in submaximal aerobic capacity and PA levels that were not apparent in the Fiuza-Luces et al. [29] study. Primary differences observed in the exercise programming by Fiuza-Luces et al. [29] included a three session per week protocol run for 16 weeks, 60–70 min training sessions, and a continuous bout of aerobic activity. Interestingly, there were no major differences between strength protocols in the two studies (all major muscle groups, 2–3 sets of 8–15 repetitions with 1–2 min rest between), although there was no information regarding resistance intensity in the study by Fiuza-Luces et al. [29]. As such, we can only speculate as to whether the adherence issues experienced here affected our strength results. However, Piscione et al. [49] also failed to see improvements in global body strength following a 12 week intervention. On the other hand, San Juan et al. [30] observed significant improvements in global strength, functional mobility and VO_2peak_ following 16 weeks of intra-hospital exercise training with pediatric leukemia patients. Once again, details regarding resistance intensity were not reported, however the remaining strength protocols were similar to what was implemented here (11 exercises for all major muscle groups, 1 set of 8–15 repetitions with 1–2 min rest in between) [30]. Aerobic activities similarly worked participants at 50–70% of their HR maximum for 10–30 min, although running and aerobic games were also part of the protocol implemented by San Juan et al. [30]. These varied exercise intervention results highlight the complexity of this population and suggest that confounding variables such as cancer treatment and associated side-effects may affect acquisition of exercise benefits irrespective of training protocol [5, 10, 56].

The primary limitations of this study were the small and heterogeneous study population, the lack of healthy control group and the variable exercise attendance. Further, with regards to PA measures, accelerometers can only accurately record unweighted, full-body motion. Therefore, participants who undertook any stationary, isolated and/or weight-bearing exercise such as squats or bicep-curls would have had seemingly lower levels of PA compared to those who completed an activity such as running.

This study demonstrates that implementation of exercise programs in AYA survivors of pediatric oncology related cerebral insult can be challenging, albeit overall feasible. Although we saw reduced attendance at the third, weekend exercise session (only four participants consistently attended all three sessions throughout the course of the study) many participants noted that they would have attended the class regularly if it wasn’t scheduled on a Saturday (attendance among the remaining participants varied based on whether work or social events had been planned for the weekend). Further, the primary reported barrier to regular participation among the non-compliant participants was conflicting work and university timetables. This indicates the importance of considering participants’ schedules and lifestyles when planning future interventions. Importantly, participants noted that they enjoyed having individualized programs that they could perform in a group setting and greatly valued their relationships with the trainers. Future research should aim to determine whether shorter exercise programs can confer the same benefits while maintaining participant compliance.

In conclusion, we have demonstrated that long-term exercise significantly improves vascular function and hence decreases the risk of cardiac events in AYA survivors of pediatric oncology related cerebral insult. These effects occurred despite a lack of change in fat mass and peak aerobic capacity. In addition, sedentary break time increased as a result of exercise, translating into a further amelioration of CVD risk. Finally, exercise may aid performance of ADL’s and health-related behaviors via improvements in submaximal aerobic capacity and local strength. While our results need to be interpreted with some caution due to the small and heterogeneous sample, it is assuring to see such significant findings in this limited population; thus, there is much promise for the success of future interventions utilizing larger and more representative populations.

## Supporting information

S1 Table Comparison data for benign and malignant tumor survivors. (DOCX)
