# Supplementary material for: Exercise training improves vascular function and secondary health measures in survivors of pediatric oncology related cerebral insult
Source: PLoS One. 2018 Aug 9;13(8):e0201449. doi: 10.1371/journal.pone.0201449 (PMC6084859; doi:10.1371/journal.pone.0201449)
Supplement: S1 Table — (DOCX) [file pone.0201449.s001.docx]

**S1 Table. Comparison data for benign and malignant tumor survivors.**

|  | Baseline | | Following  Regular Care | | Following  Exercise | | | Time | Nature of Tumor | Interaction |
| --- | --- | --- | --- | --- | --- | --- | --- | --- | --- | --- |
|  | Benign | Malignant | Benign | Malignant | | Benign | Malignant | *P* value | *P* value | *P* value |
| Heart Rate and Blood Pressure |  |  |  |  | |  |  |  |  |  |
| N | 8 | 5 | 8 | 5 | | 8 | 5 |  |  |  |
| Systolic Blood Pressure (mmHg) | 115 ± 10 | 105 ± 7 | 116 ± 11 | 108 ± 10 | | 117 ± 14 | 114 ± 15 | 0.093 | 0.259 | 0.378 |
| Diastolic Blood Pressure (mmHg) | 66 ± 3 | 61 ± 2 | 64 ± 5 | 61 ± 3 | | 63 ± 6 | 62 ± 7 | 0.737 | 0.109 | 0.481 |
| Mean Arterial Pressure (mmHg) | 85 ± 6 | 77 ± 3 | 82 ± 7 | 78 ± 3 | | 84 ± 9 | 82 ± 9 | 0.548 | 0.182 | 0.406 |
| Heart Rate (bpm) | 71 ± 9 | 71 ± 8 | 71 ± 12 | 78 ± 11 | | 74 ± 17 | 79 ± 16 | 0.488 | 0.440 | 0.406 |
| Endothelial Function |  |  |  |  | |  |  |  |  |  |
| N | 8 | 5 | 8 | 5 | | 8 | 5 |  |  |  |
| Baseline Diameter (cm) | 3.42 ± 0.57 | 3.08 ± 0.67 | 3.37 ± 0.48 | 3.31 ± 0.88 | | 3.37 ± 0.54 | 3.24 ± 0.88 | 0.686 | 0.623 | 0.373 |
| Peak Diameter (cm) | 3.76 ± 0.57 | 3.33 ± 0.63 | 3.72 ± 0.46 | 3.59 ± 0.87 | | 3.75 ± 0.53 | 3.60 ± 0.85 | 0.420 | 0.504 | 0.301 |
| Delta Diameter (cm) | 0.34 ± 0.07 | 0.26 ± 0.09 | 0.36 ± 0.06 | 0.28 ± 0.05 | | 0.37 ± 0.08^†∆^ | 0.35 ± 0.06^†∆^ | 0.002* | 0.123 | 0.131 |
| Delta Percent (%) | 10.13 ± 2.48 | 8.83 ± 3.76 | 11.06 ± 2.62 | 9.19 ± 2.53 | | 11.41 ± 3.53^†^ | 11.67 ± 3.63^†^ | 0.018* | 0.550 | 0.284 |
| Time to Peak (min) | 1.03 ± 0.49 | 0.65 ± 0.13 | 1.22 ± 0.55 | 0.97 ± 0.20 | | 0.82 ± 0.31 | 0.63 ± 0.10 | 0.099 | 0.018* | 0.847 |
| Accelerometer Data |  |  |  |  | |  |  |  |  |  |
| N | 8 | 5 | 6 | 3 | | 6 | 4 |  |  |  |
| Wear Time (min/day) | 843.44 ± 52.31 | 840.92 ± 99.98 | 741.28 ± 34.43° | 774.48 ± 9.50° | | 766.52 ± 69.39^†^ | 769.56 ± 6.35^†^ | 0.010* | 0.674 | 0.757 |
| Sedentary Breaks | 61.42 ± 20.69 | 64.45 ±20.51 | 45.61 ± 8.88 | 60.69 ± 17.25 | | 69.76 ± 17.20 | 64.89 ± 7.07 | 0.150 | 0.608 | 0.378 |
| Counts (per min) | 162.30 ± 130.06 | 170.16 ± 128.79 | 94.02 ± 17.53 | 141.13 ± 90.85 | | 170.59 ± 30.17 | 100.93 ± 38.45 | 0.310 | 0.918 | 0.202 |
| Sedentary Time (%) | 78.38 ± 8.45 | 78.04 ± 9.10 | 83.67 ± 2.43 | 79.50 ± 8.00 | | 75.18 ± 2.64 | 80.13 ± 2.75 | 0.225 | 0.965 | 0.182 |
| Light Intensity PA (%) | 19.01 ± 6.33 | 19.68 ± 7.80 | 15.61 ± 2.31 | 18.78 ± 7.07 | | 22.32 ± 3.41 | 18.99 ± 1.93 | 0.264 | 0.951 | 0.296 |
| Moderate Intensity PA (%) | 2.49 ± 3.36 | 1.78 ± 0.53 | 0.62 ± 0.30 | 1.34 ± 0.52 | | 2.38 ± 1.29 | 0.86 ± 0.83 | 0.424 | 0.546 | 0.438 |
| Vigorous Intensity PA (%) | 0.13 ± 0.20 | 0.51 ± 0.88 | 0.10 ± 0.11 | 0.40 ± 0.65 | | 0.12 ± 0.09 | 0.03 ± 0.05 | 0.188 | 0.384 | 0.185 |
| Anthropometry |  |  |  |  | |  |  |  |  |  |
| N | 8 | 5 | 8 | 5 | | 8 | 5 |  |  |  |
| Height (cm) | 170.5 ± 11.8 | 155.1 ± 6.9 | 170.3 ± 11.9 | 155.3 ± 6.9 | | 169.9 ± 12.4 | 154.9 ± 6.8 | 0.227 | 0.027* | 0.697 |
| Body Mass (kg) | 80.64 ± 21.73 | 47.41 ± 12.09 | 80.24 ± 20.63 | 47.64 ± 13.09 | | 81.51 ± 19.91 | 47.24 ± 13.34 | 0.806 | 0.008* | 0.643 |
| Body Mass Index (kg/m^2^) | 28.1 ± 8.5 | 19.6 ± 4.5 | 28.1 ± 8.7 | 19.6 ± 4.9 | | 28.8 ± 9.0 | 19.5 ± 5.0 | 0.572 | 0.065 | 0.470 |
| Body Composition |  |  |  |  | |  |  |  |  |  |
| N | 8 | 5 | 8 | 5 | | 8 | 5 |  |  |  |
| Total Fat Mass (kg) | 29.63 ± 16.35 | 14.36 ± 6.48 | 29.38 ± 16.55 | 16.88 ± 8.30 | | 29.31 ± 16.94 | 13.50 ± 7.06 | 0.407 | 0.090 | 0.396 |
| Total Percent Fat (%) | 37.49 ± 12.48 | 31.46 ± 8.90 | 37.43 ± 13.55 | 32.38 ± 10.48 | | 36.38 ± 13.94 | 28.80 ± 10.37 | 0.138 | 0.382 | 0.568 |
| VAT Mass (g) | 825.70 ± 914.61 | 270.69 ± 196.46 | 824.27 ± 742.55 | 349.49 ± 224.21 | | 501.84 ± 290.54 | 252.40 ± 119.14 | 0.387 | 0.128 | 0.540 |
| VAT Volume (cm^3^) | 875.52 ± 969.32 | 287.07 ± 208.29 | 873.78 ± 786.95 | 370.84 ± 237.72 | | 532.13 ± 308.07 | 267.60 ± 126.00 | 0.387 | 0.128 | 0.540 |
| Total Lean Body Mass (kg) | 47.05 ± 10.93 | 30.34 ± 8.25 | 47.19 ± 12.01 | 32.09 ± 9.20 | | 48.67 ± 11.27 | 31.31 ± 8.65 | 0.232 | 0.018* | 0.331 |
| Muscular Strength (kg) |  |  |  |  | |  |  |  |  |  |
| N | 8 | 5 | 8 | 5 | | 8 | 5 |  |  |  |
| Lateral Pull Downs | 48 ± 16 | 29 ± 12 | 45 ± 20 | 30 ± 15 | | 44 ± 22 | 33 ± 12 | 0.792 | 0.139 | 0.199 |
| Bicep Curl | 8.0 ± 3.0 | 6.0 ± 2.0 | 9.0 ± 3.0 | 7.0 ± 4.0 | | 9.5 ± 3.0^†∆^ | 8.0 ± 4.0^†∆^ | 0.013* | 0.280 | 0.864 |
| Muscular Endurance (60 s) |  |  |  |  | |  |  |  |  |  |
| N | 8 | 5 | 8 | 5 | | 8 | 5 |  |  |  |
| Squats | 30 ± 12 | 38 ± 19 | 27 ± 11 | 34 ± 11 | | 28 ± 7 | 40 ± 16 | 0.361 | 0.198 | 0.563 |
| Sit-ups | 24 ± 7 | 29 ± 15 | 28 ± 9 | 27 ± 16 | | 26 ± 10 | 32 ± 13 | 0.638 | 0.556 | 0.204 |
| Push-ups | 23 ± 10 | 23 ± 16 | 23 ± 9 | 18 ± 11 | | 23 ± 11^†^ | 34 ± 18^†^ | 0.039* | 0.779 | 0.031* |
| Aerobic Capacity (3 min Stage) |  |  |  |  | |  |  |  |  |  |
| N | 8 | 5 | 8 | 5 | | 8 | 5 |  |  |  |
| Rating Perceived Exertion | 8 ± 2 | 8 ± 1 | 8 ± 2 | 7 ± 1 | | 7 ± 1 | 8 ± 2 | 0.400 | 0.971 | 0.179 |
| Submaximal Heart Rate (bpm) | 103 ± 7 | 106 ± 14 | 100 ± 14 | 107 ± 14 | | 97 ± 14 | 103 ± 14 | 0.360 | 0.390 | 0.790 |
| Minute Ventilation (L·min^-1^) | 26.35 ± 9.80 | 19.19 ± 4.25 | 19.83 ± 6.32° | 13.62 ± 2.75° | | 22.30 ± 5.38^∆^ | 18.47 ± 2.17^∆^ | 0.007* | 0.076 | 0.617 |
| Respiratory Exchange Ratio | 0.83 ± 0.35 | 0.75 ± 0.04 | 0.62 ± 0.06 | 0.60 ± 0.07 | | 0.71 ± 0.04 | 0.73 ± 0.06 | 0.062 | 0.535 | 0.582 |
| VO_2_ (L·min^-1^) | 1.33 ± 0.37 | 0.98 ± 0.20 | 1.01 ± 0.42° | 0.56 ± 0.16° | | 0.98 ± 0.30^†^ | 0.68 ± 0.15^†^ | 0.007* | 0.018* | 0.655 |
| VO_2_ (ml·kg^-1^·min^-1^) | 17.03 ± 4.51 | 16.24 ± 0.98 | 12.34 ± 2.46° | 11.58 ± 4.17° | | 12.78 ± 2.48^†∆^ | 14.69 ± 2.43^†∆^ | 0.018* | 0.905 | 0.440 |
| Aerobic Capacity (6 min Stage) |  |  |  |  | |  |  |  |  |  |
| N | 8 | 5 | 8 | 5 | | 8 | 5 |  |  |  |
| Rating Perceived Exertion | 10 ± 2 | 9 ± 2 | 9 ± 2 | 8 ± 2 | | 8 ± 2^†^ | 7 ± 1^†^ | 0.024* | 0.213 | 0.682 |
| Submaximal Heart Rate (bpm) | 135 ± 13 | 141 ± 24 | 129 ± 20 | 137 ± 21 | | 119 ± 15^†∆^ | 126 ± 17^†∆^ | 0.002* | 0.474 | 0.960 |
| Minute Ventilation (L·min^-1^) | 31.32 ± 8.83 | 23.86 ± 6.00 | 26.79 ± 9.11° | 19.25 ± 2.75° | | 25.83 ± 6.84^†^ | 19.84 ± 2.63^†^ | 0.014* | 0.075 | 0.872 |
| Respiratory Exchange Ratio | 0.90 ± 0.37 | 0.80 ± 0.04 | 0.67 ± 0.10 | 0.66 ± 0.08 | | 0.77 ± 0.03 | 0.79 ± 0.06 | 0.072 | 0.632 | 0.589 |
| VO_2_ (L·min^-1^) | 1.46 ± 0.34 | 1.17 ± 0.29 | 1.34 ± 0.45° | 0.84 ± 0.21° | | 1.13 ± 0.29^†∆^ | 0.75 ± 0.21^†∆^ | 0.000* | 0.039* | 0.324 |
| VO_2_ (ml·kg^-1^·min^-1^) | 18.52 ± 2.49 | 19.21 ± 2.04 | 16.60 ± 2.36° | 16.75 ± 2.94° | | 14.74 ± 1.42^†^ | 16.11 ± 2.80^†^ | 0.002* | 0.425 | 0.776 |
| Aerobic Capacity (9 min Stage) |  |  |  |  | |  |  |  |  |  |
| N | 8 | 5 | 8 | 5 | | 8 | 5 |  |  |  |
| Rating Perceived Exertion | 11 ± 2 | 11 ± 2 | 10 ± 2 | 10 ± 2 | | 10 ± 2 | 8 ± 3 | 0.137 | 0.486 | 0.798 |
| Submaximal Heart Rate (bpm) | 160 ± 5 | 161 ± 19 | 155 ± 10 | 158 ± 21 | | 149 ± 5^†^ | 155 ± 19^†^ | 0.006* | 0.646 | 0.614 |
| Minute Ventilation (L·min^-1^) | 39.67 ± 13.04 | 29.21 ± 7.22 | 31.84 ± 10.08° | 22.92 ± 3.51° | | 30.33 ± 7.96^†^ | 23.17 ± 4.59^†^ | 0.001* | 0.081 | 0.713 |
| Respiratory Exchange Ratio | 1.02 ± 0.55 | 0.85 ± 0.06 | 0.73 ± 0.08 | 0.71 ± 0.08 | | 0.81 ± 0.02 | 0.79 ± 0.04 | 0.145 | 0.495 | 0.572 |
| VO_2_ (L·min^-1^) | 1.77 ± 0.45 | 1.40 ± 0.39 | 1.58 ± 0.48° | 0.78 ± 0.37° | | 1.32 ± 0.41^†^ | 0.84 ± 0.24^†^ | 0.000* | 0.017* | 0.161 |
| VO_2_ (ml·kg^-1^·min^-1^) | 22.58 ± 4.03 | 22.86 ± 3.04 | 19.38 ± 2.07° | 19.75 ± 3.63° | | 17.03 ± 2.53^†∆^ | 17.74 ± 2.47^†∆^ | 0.000* | 0.691 | 0.981 |
| Aerobic Capacity (Peak) |  |  |  |  | |  |  |  |  |  |
| N | 8 | 5 | 8 | 5 | | 8 | 5 |  |  |  |
| Rating Perceived Exertion | 16 ± 2 | 18 ± 1 | 16 ± 2 | 16 ± 2 | | 17 ± 3 | 17 ± 4 | 0.323 | 0.658 | 0.513 |
| Maximal Heart Rate (bpm) | 185 ± 6 | 181 ± 16 | 180 ± 8 | 179 ± 25 | | 179 ± 9 | 183 ± 22 | 0.280 | 0.971 | 0.075 |
| Minute Ventilation (L·min^-1^) | 81.79 ± 34.86 | 59.40 ± 18.58 | 71.17 ± 23.20 | 48.46 ± 16.43 | | 70.65 ± 24.76 | 65.96 ± 20.03 | 0.086 | 0.232 | 0.128 |
| Respiratory Exchange Ratio | 1.00 ± 0.07 | 0.96 ± 0.11 | 0.91 ± 0.08 | 0.91 ± 0.12 | | 1.02 ± 0.08^∆^ | 1.01 ± 0.09^∆^ | 0.006* | 0.683 | 0.768 |
| VO_2peak_ (L·min^-1^) | 3.01 ± 1.13 | 2.26 ± 0.72 | 2.91 ± 1.04 | 1.73 ± 0.84 | | 2.96 ± 1.09 | 1.80 ± 0.89 | 0.085 | 0.084 | 0.254 |
| VO_2peak_ (ml·kg^-1^·min^-1^) | 39.30 ± 15.13 | 37.22 ± 7.18 | 38.03 ± 13.22 | 36.28 ± 10.94 | | 38.84 ± 14.50 | 38.03 ± 12.92 | 0.450 | 0.835 | 0.833 |
| VO_2peak_ (ml·LBM^-1^·min^-1^) | 61.91 ± 12.45 | 59.74 ± 5.26 | 63.06 ± 8.88 | 57.03 ± 13.84 | | 59.86 ± 10.74 | 55.67 ± 12.82 | 0.276 | 0.495 | 0.610 |

VAT, visceral adipose tissue; LBM, lean body mass

*Denotes statistical significance (p ≤ 0.05) for given effect using Mixed-Model ANOVA

°Denotes statistical significance (p ≤ 0.05) between Baseline and Regular Care using post-hoc paired t-tests

†Denotes statistical significance (p ≤ 0.05) between Baseline and Exercise using post-hoc paired t-tests

∆Denotes statistical significance (p ≤ 0.05) between Regular Care and Exercise using post-hoc paired t-tests
